# Nonoperative management for patients with grade IV blunt hepatic trauma

**DOI:** 10.1186/1749-7922-7-S1-S8

**Published:** 2012-08-22

**Authors:** Thiago Messias Zago, Bruno Monteiro Tavares Pereira, Thiago Rodrigues Araujo Calderan, Mauricio Godinho, Bartolomeu Nascimento, Gustavo Pereira Fraga

**Affiliations:** 1 Rua Alexander Fleming, 181 Zip code: 13.083-970, Cidade Universitaria “Prof. Zeferino Vaz, Campinas – SP, Brazil; 2 2075 Bayview Ave., Room B5 12, Toronto, Ontario, M4N 3M5 Canada

## Abstract

**Introduction:**

The treatment of complex liver injuries remains a challenge. Nonoperative treatment for such injuries is increasingly being adopted as the initial management strategy. We reviewed our experience, at a University teaching hospital, in the nonoperative management of grade IV liver injuries with the intent to evaluate failure rates; need for angioembolization and blood transfusions; and in-hospital mortality and complications.

**Methods:**

This is a retrospective analysis conducted at a single large trauma centre in Brazil. All consecutive, hemodynamically stable, blunt trauma patients with grade IV hepatic injury, between 1996 and 2011, were analyzed. Demographics and baseline characteristics were recorded. Failure of nonoperative management was defined by the need for surgical intervention. Need for angioembolization and transfusions, in-hospital death, and complications were also assessed

**Results:**

Eighteen patients with grade IV hepatic injury treated nonoperatively during the study period were included. The nonoperative treatment failed in only one patient (5.5%) who had refractory abdominal pain. However, no missed injuries and/or worsening of bleeding were observed during the operation. None of the patients died nor need angioembolization. No complications directly related to the liver were observed. Unrelated complications to the liver occurred in three patients (16.7%); one patient developed a tracheal stenosis (secondary to tracheal intubation); one had pleural effusion; and one developed an abscess in the pleural cavity. The hospital length of stay was on average 11.56 days.

**Conclusions:**

In our experience, nonoperative management of grade IV liver injury for stable blunt trauma patients is associated with high success rates without significant complications.

## Introduction

The treatment of complex liver injuries remains a challenge for surgeons despite the last decade’s advances in diagnostic and therapeutic techniques. The mortality rate for liver injuries grade IV (parenchymal disruption involving 25–75% of hepatic lobe or 1–3 Coinaud’s segments in a single lobe) in the literature varies from 8% to 56%. [1-4].

The nonoperative treatment for such injuries in hemodynamically stable patients with blunt abdominal trauma admitted with no signs of peritonitis is being progressively more utilized as the initial therapeutic approach in many designated trauma centers. Although some studies have demonstrated that the nonoperative treatment is safe for selected patients, many surgeons still choose to operate high-grade hepatic injuries solely according to the grade of the injury [5-8].

One of the most significant advances in the management of trauma patients in recent years was the introduction of Computed Tomography (CT) scan for stable patients. The recommendations on the use of CT for hemodynamically stable patients are well established, as outlined by the manual of the Advanced Trauma Life Support (ATLS^®^) of the American College of Surgeons. CT scan allows detection and classification of hepatic lesions and excludes the presence of associated injuries; especially injuries to hollow viscera, although in some cases it underestimates the findings. CT scan, due to its high sensitivity, specificity and accuracy, is an important screening and diagnostic tool for intra-abdominal injuries in hemodynamically stable patients; patients with altered level of consciousness; and those with difficult clinical examination or associated pelvic fractures [9-12].

The goal of this study was to determine the effectiveness of nonoperative management of grade IV liver injuries evaluating failure rates; need for angioembolization and blood transfusions; and in-hospital morbidity and mortality.

## Methods

Our University teaching hospital is one of the referral trauma centers in a metropolitan area of approximately 2.8 million people. This study included patients admitted to our trauma center from 1996 through 2011. The study protocol was reviewed and approved by our institution’s research ethics board.

Patients were eligible for this analysis if they were adult (15 years or more); sustained grade IV hepatic injury, classified according to the American Association for the Surgery of Trauma Organ Injury Scale (grade IV hepatic trauma corresponds to parenchymal disruption involving 25–75% of hepatic lobe or 1–3 Coinaud’s segments in a single lobe) [1]; and were initially managed nonoperatively as per our hospital guidelines for hepatic injury. We excluded all patients who did not meet the aforementioned inclusion criteria.

All patients were initially resuscitated in accordance to the Advanced Trauma Life Support (ATLS^®^) and were submitted to CT scan examination. Selection criteria for nonoperative liver injuries management were hemodynamic stability after initial resuscitation with crystalloid and no need for blood transfusion, absence of clinical signs of peritonitis, and no bowel injuries shown on CT scan.

The nonoperative treatment protocol adopted in our trauma division is described in Table 1.

**Table 1:** Protocol of nonoperative management in AAST-OIS grade IV blunt hepatic trauma.

Protocol of nonoperative management in AAST-OIS grade IV blunt hepatic trauma - Division of Trauma Surgery - University of Campinas
**Criteria for patient selection:** 1- Abdominal blunt trauma 2- Hemodynamic stability after initial resuscitation with no need for blood: a. Systemic blood pressure > 90 mmHg b. Initial hemoglobin level > 8 3- Evaluation by Computed Tomography with: a. Absence of associated injuries on hollow viscus and pneumoperitonium b. Absence of contrast blush (evidence of active arterial bleeding is indication for angiography and embolization) 4- Clinical evaluation with no signs of peritonitis

**Monitorization of patients undergoing nonoperative management:** 1- Hemoglobin/ Hematocrit measurement every 6 hours or more frequently if any clinical deterioration 2- ABG measurements every 6 hours or more frequently if any clinical deterioration 3- ICU (Intensive Care Unit)

**Criteria for failure of nonoperative management:** 1- Need for surgical intervention determined by: a. Hemodynamic instability b. Failure of angioembolization to control active bleeding c. Progressive fall of hemoglobin/ hematocrit levels with recurrent blood transfusion d. Clinical signs of peritonitis

Until March 2009 helical CT scan was used as a diagnostic tool. After this period, multi-slice CT became routine for all admitted trauma patients in our hospital. For the CT scan evaluation, the patient must be hemodynamically stable, or remain stable after adequate fluid replacement. According to this protocol, Glasgow Coma Score wasn’t an exclusion criterion. The presence of contrast extravasation has usually indicated embolization through arteriography prior to surgery indication.

### Study variables and outcome measures

Age, gender, mechanism of injury, systolic blood pressure (SBP), Revised Trauma Score (RTS), Injury Severity Score (ISS), CT scan findings, presence of associated abdominal injuries, need for surgical intervention, need for blood transfusions, complications related to liver (re-bleeding of the liver, biliary fistula, biliar peritonitis, liver abscess and intra-abdominal abscess) and non-liver related complications (pneumonia, empyema, atelectasis, Adult Respiratory Distress Syndrome, kidney failure, intestinal fistulae, urinary tract infections, sepsis and brain injury), mortality and length of stay in the hospital, were analyzed [13,14].

### Statistical analysis

Discrete variables are summarized as frequency and percentages. Summary data for continuous variables is presented as means and standard deviations, or medians and ranges depending on the distribution.

## Results

During the study period, 754 patients with hepatic trauma were admitted in our service. This total included 294 (39%) patients with blunt hepatic trauma. Eighty patients (27.2%) of this total met the criteria and were treated nonoperatively. Eighteen (22.5%) out of these 80 patients were classified as having a grade IV hepatic injury; and thus constitute the study cohort. Of the 18 admitted patients with AAST-OIS grade IV blunt hepatic trauma, six patients (33.3%) were women and 12 patients (66.7%) were men. The mean age of patients was 34.22 ± 13.02 years, ranging from 20 to 59 years.

The mechanisms of injury are distributed as follows: 11 patients were involved in motor vehicle crashes; 7 (38.9%) in motorcycle collisions; and 4 (22.2%) in small utility car crashes. Two (11.1%) were pedestrians hit by a car and 5 patients (27.8%) suffered other types of blunt trauma.

The mean systolic blood pressure on admission was 116.76 ± 28.33 mmHg. The only patient admitted with hypotension remained stable after 2000 ml crystalloid infusion. The mean Revised Trauma Score was 7.60 ± 0.58. The average Injury Severity Score of these patients was 24.11 ± 8.73.

Twelve patients (66.7%) required blood transfusion, with a mean of 2.26 ± 1.57 packed red blood cells per patient.

Additional abdominal injuries were found in four patients (22.2%). Kidney was the most affected organ (all 4 patients), and the spleen was affected in one patient. None of the patients developed complications related to the liver injury. Complications unrelated to the liver occurred in 3 patients (16.7%); 1 developed a tracheal stenosis (secondary to tracheal intubation); 1 had a pleural effusion; and 1 an abscess in the pleural cavity. Patient characteristics evaluated are described in Table 2.

**Table 2:** Evaluated aspects of patients with grade IV blunt hepatic trauma undergoing nonoperative management.

**Demographics and baseline characteristics** Aspect evaluated	**N=18** Frequence / mean (n/ SD)
Male	66.7% (12)
Age	34 (± 13)
Systolic Blood Pressure on admission	117 (± 28)
RTS	7.6 (± 0.58)
ISS	24 (± 9)
Blood transfusion	66.7% (12)
Packed red blood cell transfused	2.26 ± 1.57
Associated abdominal injuries	22.2% (4)

**Figure 1:**
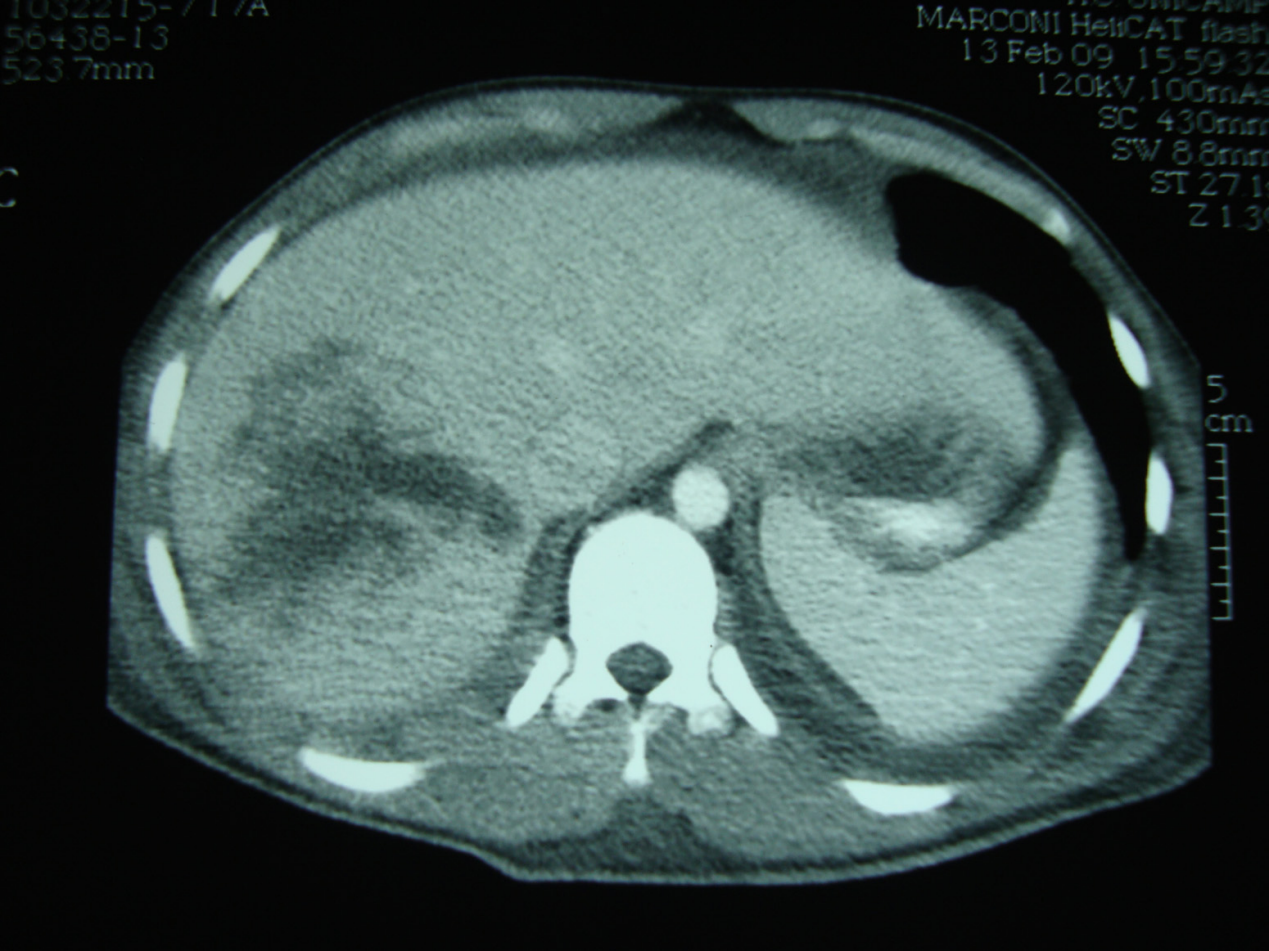
Pedestrian hit by a car; multislice CT showing abdominal free fluid and intraparenchymal hematoma in the right lobe (grade IV hepatic injury), no blush of contrast in the arterial phase**.**

**Figure 2:**
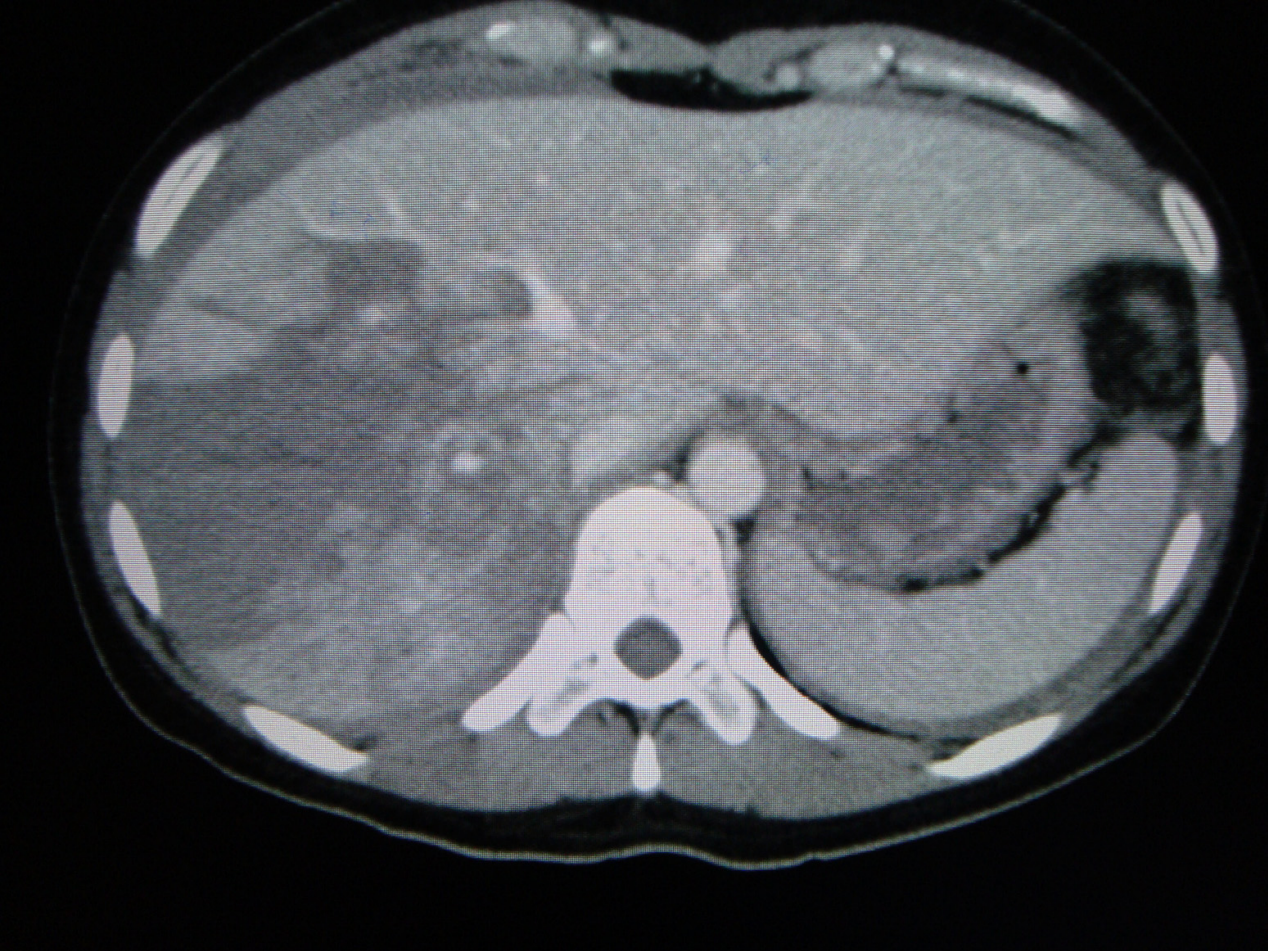
Bicycle crash; multisclice CT showing the presence of abdominal free fluid, with intraparenchymal hematoma in the right lobe (grade IV hepatic injury), no blush of contrast in the arterial phase.

Regarding the CT scan findings, seven patients (38.8%) had isolated hepatic injury with perihepatic fluid and 11 patients (61.1%) had liver injury and free fluid in the abdominal cavity (Figures 1 and 2). Ten patients (55.5%) had helical CT evaluation while 8 (44.5%) had multi-slice CT scans. Six patients (33.3%) had repeated follow-up scans, on average 5 days after the initial CT. None of the follow-up CTs demonstrated progression of the injury. Nonoperative management failed in a single patient (5.5%) that had a progression of the free fluid (hemoperitoneum) in the abdomen along with peritonitis. The patient was operated 4 days after admission when a large hemoperitoneum was found but no active bleeding from the liver. Thus nonoperative hepatic trauma management as per our protocol resulted in an overall success rate of 94.5%. No patient died and the mean hospital stay was 11.56 ± 5.3 days (Table 3).

**Table 3:** Outcome of patients with grade IV blunt hepatic trauma undergoing nonoperative management.

Outcome Aspect evaluated	N=18 Frequence / mean (n/SD)
Complications related to the liver	0
Non -liver related complications	16.7% (3)
Failure of nonoperative management	5.5% (1)
In-hospital Mortality	0
Length of hospital stay	11.56 ± 5.3

## Discussion

Since 1980 several studies have proposed that nonoperative treatment of blunt liver injuries be considered the treatment of choice for patients with hemodynamic stability. The great capacity of the liver for regenerating, the pattern of venous bleeding, and the high rate of spontaneous hemostasis, may explain and be responsible for high success rates associated with nonoperative treatment. Pachter et al, in a multicenter study with 13 Level I Trauma Centers in the USA, reported a 98.5% rate of success in nonoperative treatment for selected patients [7,8,12,15-18].

Severe liver injuries (grade III, IV and V) have higher morbidity and mortality. In a study with 170 patients with hepatic trauma, Rizoli et al observed a total of 10 deaths, all with grade IV and V injuries. Many surgeons choose to operate complex lesions of the liver even in patients admitted with hemodynamic stability, fearing a possible rebleeding of liver injury. It is known that the liver rebleeding in patients admitted with hemodynamic stability and with no blush on CT scan, is a rare event [2,6,16,19].

Patients admitted with severe liver injuries tend to be more critical. The average ISS of patients in this study was 24.1. Kozar et al found an average of ISS 28 for patients with grade IV blunt hepatic trauma. In other studies involving patients with blunt or penetrating liver trauma with grade IV and V injuries, submitted to surgical treatment or non-surgical, the average ISS was 25, 33, 34 and 36 respectively [2,6,20-22].

None of the patients in our study died, in agreement with other studies showing that nonoperative treatment for grade IV blunt hepatic trauma is safe for selected patients [5,22].

In this study we observed that none of the 18 patients developed any complications related to the liver and three patients developed non-liver related complications. Kozar et al found complications in 19 of 92 patients (21%) with grade IV injuries treated nonoperatively. Of these patients, less than a half needed some kind of surgical intervention. Duane et al reported a complication rate of 0% for patients with grade IV blunt liver injury that did not undergo surgery or angioembolization [6,22].

Only one of the 18 patients studied herein required surgical conversion secondary to abdominal pain, showing a success rate of 94.5% of nonoperative treatment. In a study with patients with grades III and IV hepatic trauma Coimbra et al, related that 22% of patients undergoing nonoperative treatment needed surgical intervention. In another study with 230 patients with grades III, IV and V blunt hepatic trauma treated nonoperatively, Kozar et al had 12 patients (5.2%) who failed with nonoperative management and required surgical intervention [5,6].

The abdominal CT scan is the diagnostic modality of choice for hemodynamically stable patients with suspected abdominal injuries. CT scan has some advantage over ultrasound exam. CT is less operator-dependent and is not limited by the abdominal wall, subcutaneous emphysema, obesity or intestinal distention. CT is very important to diagnose abdominal injuries in patients with neurological damage, since physical examination is feasible in no more than 16% of these patients [12,22-27].

CT scan allows visualization of hemoperitoneum, one of the most obvious signs of the presence of abdominal injury. Usually the hemoperitoneum is seen in the Morison pouch, perihepatic space and in the right paracolic gutter and is reabsorbed after 5 to 10 days after injury. The amount of hemoperitoneum have previously been considered an indicator of liver trauma severity, but some recent studies have indicated that the amount of hemoperitoneum does not correlate with failure of nonoperative management [12,17,24,28,29]. Besides hemoperitoneum, CT allows the visualization of contusions, subcapsular hematomas, intraparenchymal hematomas and lacerations to the liver parenchyma [30,31].

An important role of the CT scan is to detect active extravasation of contrast, indicating the presence of active bleeding. With this information, an angiography should be performed even in hemodinamically stable patients due to the risk of bleeding and subsequent failure of the nonoperative management. Angiographic embolization is a safe strategy in the management of hepatic arterial hemorrhage in patients with blunt trauma. It was demonstrated to reduce the amount of transfusions, the need for further liver-related surgeries and the mortality in high-grade liver injuries. Almost all patients in this series were evaluated by helical CT scan, which has a low accuracy to identify extravasation of contrast. This explains the fact that no patient underwent angiographic embolization in the present study [21,32-36].

Besides the diagnostic capacity, CT also has an important role in monitoring patients treated nonoperatively. In this study, the follow-up CT did not have an important role. Six patients were submitted to follow-up CT, which never demonstrated worsening in the injuries or contributed for the indication of any intervention. In a study with 74 patients with grade IV blunt liver trauma treated nonoperatively and with repeated performance of CT, only three patients required another therapeutic procedure. Of these three patients, two underwent angiography and one drainage of a bilioma. However, these three patients had strong clinical signs of changes in the clinical course as tachycardia, abdominal pain and elevated enzymes. Another study concluded that repeated CT scan matters in patients with clinical deterioration and signs of peritonitis or sepsis [18,24,37,38].

## Conclusions

In our experience, the nonoperative treatment can be performed in trauma centers with protocols in place; 24-hour operating rooms; trained surgical teams; blood banks; critical care support; and image diagnosing methods available, such as mult-islide or helical CT scan. Although AAST-OIS grade IV blunt hepatic trauma patients are critical, nonoperative approach can be adopted in hemodynamically stable patients safely and with high success rates.

## Competing interests

***Sources of funding*:** Fundação de Amparo à Pesquisa do Estado de São Paulo (FAPESP). Grant number 12698/2010.

## Authors’ contributions

***TMZ*** participated in the conception, design and intellectual content, collection, analysis and interpretation of data. ***BMTP*** participated in the intellectual content; revision of the manuscript, figures and tables. ***TRAC*** participated in the revision of the manuscript, figures and tables. ***MG*** participated in the revision of the manuscript, figures and tables. ***BN*** participated in the revision of the manuscript, figures and tables. ***GPF*** had overall responsibility for the study including conception, design and intellectual content, collection, analysis and interpretation of data.

## Authors’ information

***Thiago Messias Zago***. Medical student of Faculty of Medical Sciences (FCM) – University of Campinas (Unicamp).

***Bruno Monteiro Tavares Pereira***. Assistant Surgeon of Division of Trauma Surgery, FCM - Unicamp.

***Thiago Rodrigues Araujo Calderan***. Assistant Surgeon of Division of Trauma Surgery, FCM - Unicamp.

***Mauricio Godinho***. Assistant Surgeon of Division of Trauma Surgery, FCM - Unicamp.

***Bartolomeu Nascimento***. Fellow, Trauma Program, Sunnybrook Health Sciences Centre, University of Toronto and Visiting Professor of the Division of Trauma Surgery, FCM - Unicamp*.*

***Gustavo Pereira Fraga***. Professor of Surgery and Coordinator of Division of Trauma Surgery, FCM - Unicamp.
